# Rituximab abrogates aquaporin-4–specific germinal center activity in patients with neuromyelitis optica spectrum disorders

**DOI:** 10.1073/pnas.2121804119

**Published:** 2022-06-06

**Authors:** Valentina Damato, Jakob Theorell, Adam Al-Diwani, Anne-Kathrin Kienzler, Mateusz Makuch, Bo Sun, Adam Handel, Deniz Akdeniz, Antonio Berretta, Sudarshini Ramanathan, Andrew Fower, Daniel Whittam, Emily Gibbons, Nicholas McGlashan, Edward Green, Saif Huda, Mark Woodhall, Jacqueline Palace, Fintan Sheerin, Patrick Waters, Maria I. Leite, Anu Jacob, Sarosh R. Irani

**Affiliations:** ^a^Oxford Autoimmune Neurology Group, Nuffield Department of Clinical Neurosciences, University of Oxford, Oxford OX3 9DU, United Kingdom;; ^b^Department of Neurology, John Radcliffe Hospital, Oxford University Hospitals, Oxford OX3 9DU, United Kingdom;; ^c^Department of Neuroscience, Università Cattolica del Sacro Cuore, Rome 00168, Italy;; ^d^Institute of Neurology, Fondazione Policlinico Universitario 'A. Gemelli' IRCCS, Rome 00168, Italy;; ^e^Department of Clinical Neurosciences, Karolinska Institutet, Stockholm 17177, Sweden;; ^f^Department of Psychiatry, University of Oxford, Oxford OX3 9DU, United Kingdom;; ^g^Translational Neuroimmunology Group, Kids Neuroscience Centre, Children’s Hospital at Westmead, Sydney NSW 2145, Australia;; ^h^Department of Neurology, The Walton Centre NHS Foundation Trust, Liverpool L9 7LJ, United Kingdom;; ^i^Department of Neuroradiology, John Radcliffe Hospital, Oxford University Hospitals, Oxford OX3 9DU, United Kingdom;; ^j^Neurological Institute, Cleveland Clinic, Abu Dhabi F9XQ, United Arab Emirates

**Keywords:** rituximab, aquaporin, autoimmunity, neuromyelitis optica, cervical lymph nodes

## Abstract

Neuromyelitis optica spectrum disorders (NMOSDs) are caused by immunoglobulin G (IgG) autoantibodies directed against the water channel aquaporin-4 (AQP4). In NMOSDs, discrete clinical relapses lead to disability and are robustly prevented by the anti-CD20 therapeutic rituximab; however, its mechanism of action in autoantibody-mediated disorders remains poorly understood. We hypothesized that AQP4-IgG production in germinal centers (GCs) was a core feature of NMOSDs and could be terminated by rituximab. To investigate this directly, deep cervical lymph node (dCLN) aspirates (*n* = 36) and blood (*n* = 406) were studied in a total of 63 NMOSD patients. Clinical relapses were associated with AQP4-IgM generation or shifts in AQP4-IgG subclasses (odds ratio = 6.0; range of 3.3 to 10.8; *P* < 0.0001), features consistent with GC activity. From seven dCLN aspirates of patients not administered rituximab, AQP4-IgGs were detected alongside specific intranodal synthesis of AQP4-IgG. AQP4-reactive B cells were isolated from unmutated naive and mutated memory populations in both blood and dCLNs. After rituximab administration, fewer clinical relapses (annual relapse rate of 0.79 to 0; *P* < 0.001) were accompanied by marked reductions in both AQP4-IgG (fourfold; *P* = 0.004) and intranodal B cells (430-fold; *P* < 0.0001) from 11 dCLNs. Our findings implicate ongoing GC activity as a rituximab-sensitive driver of AQP4 antibody production. They may explain rituximab’s clinical efficacy in several autoantibody-mediated diseases and highlight the potential value of direct GC measurements across autoimmune conditions.

## Significance

By studying paired blood and deep cervical lymph node samples from patients with neuromyelitis optica spectrum disorders, our data provide evidence for a germinal center–based generation of aquaporin-4 antibodies. Frequent serum aquaporin-4 immunoglobulin Ms (IgMs) and shifts in IgG subclasses were observed alongside preferential synthesis of aquaporin-4 IgGs and aquaporin-4–reactive B cells within lymph nodes. Both intranodal synthesis of aquaporin-4 antibodies and intranodal aquaporin-4–reactive B cells were robustly eliminated with rituximab administration. This study systematically explores lymph nodes that drain the central nervous system (CNS) in patients with CNS autoimmunity and offers a potential explanation as to why rituximab is clinically highly efficacious in autoantibody-mediated diseases despite no accompanying reduction in serum autoantibody levels.

Immunoglobulin G (IgG) autoantibodies directed against the extracellular domain of the water channel aquaporin-4 (AQP4) are directly causative in patients with neuromyelitis optica spectrum disorders (NMOSDs) (1–4). AQP4-IgGs are predominantly of the IgG1 subclass, and their major proposed pathogenic mechanism is via complement-mediated damage to the AQP4-rich astrocyte end feet (5). In NMOSDs, patient disability is accrued through discrete clinical relapses, typically affecting the spinal cord and/or optic nerve (6, 7). However, the immunobiology underlying these attacks is poorly understood, and few serum biomarkers can accurately predict relapses (8).

Traditionally, ongoing autoantibody production is considered to occur via two broadly discrete cellular pathways: continual germinal center (GC) activity versus long-lived plasma cells (LLPCs) (9). GCs are specialized microenvironments, typically located within secondary lymphoid organs, where antigen-reactive B cells diversify and mature their immunoglobulin genes via somatic hypermutation, with help from specialized lymphoid-resident T follicular helper (Tfh) cells (10). The process of somatic hypermutation is commonly observed alongside a DNA excision process known as class-switch recombination. Together, somatic hypermutation and class-switch recombination can generate high-affinity IgG responses. Autoantigen reactivity of the B cell receptor (BCR) may either arise de novo following somatic hypermutation in GCs or be originally encoded by antigen-reactive germline BCRs expressed by naive B cells (10, 11). Ongoing GC activity may be responsible for the prolonged presence of autoantibodies, such as AQP4-IgGs (9, 12). In an alternative model, LLPCs that successfully exit active GCs and acquire a bone marrow niche may autonomously persist for decades after an autoimmunizing event. These niched LLPCs are thought to secrete >90% of human serum IgG, including a variety of autoantibodies (13, 14).

To date, a series of observations suggest that GC activity may play an important role in AQP4-IgG generation. First, close correlations between serum AQP4-IgG levels and AQP4-IgG secreted in vitro by circulating B cells suggest a limited role for LLPCs in AQP4-IgG generation (12, 15). Second, the detection of circulating AQP4-reactive naive B cells identifies a source of cells that could enter GCs and are reported to share clonal relationships with the hypermutated BCRs of intrathecal AQP4-reactive plasma cells (16, 17). Next, annualized relapse rates (ARR) in NMOSDs are robustly reduced by multiple immunotherapies likely to spare nonproliferative CD20^−^ LLPCs, including the anti-CD20 monoclonal antibody rituximab (RTX) (18–20); however, likely because RTX spares the LLPCs, it does not reduce serum AQP4-IgG levels, an observation that presents a potential clinical–serological paradox in a disease with proven pathogenic autoantibodies (21, 22).

We hypothesized that the rapid clinical efficacy of RTX observed in patients with NMOSDs may be explained by its direct disruption of active GC reactions, impacting the most affinity matured, and hence pathogenic, B cells and antibodies. However, contradictory data from both human and mouse studies mean that it remains unclear whether RTX effectively depletes B cells within secondary lymphoid organs (23–25). Further, the putative role of GCs in NMOSDs has not been studied directly. In autoimmune diseases of the central nervous system (CNS), the lymphoid organs that drain meningeal lymphatics represent the most plausible anatomical site of active GCs, the deep cervical lymph nodes (dCLNs) (26).

To address these concepts, we studied 63 patients with NMOSDs as a prototypical model of an autoantibody-mediated condition. From patients seen as part of routine clinical practice in two specialist NMO centers, we identified clinical relapses in association with proxy measures of an active GC response: class-switch recombination and de novo AQP4-IgM production. Next, to directly sample the secondary lymphoid organs most likely to generate a GC response to neuronal antigens, we aspirated dCLNs from NMOSD patients. These aspirates contained intranodal AQP4-specific B cells and evidence of local, intranodal AQP4-IgG synthesis, both of which were rapidly and efficiently abrogated by RTX over a timescale consistent with clinical remission. Our data present direct insights into the immunological drivers of NMOSD, highlight the effects of RTX in a model of human autoantibody-mediated illness, and provide a platform for the direct analyses of GCs in human autoimmunity.

## Results

### Dynamics of AQP4-IgG Subclasses and AQP4-IgM Associate with Clinical Relapses.

**Fig. 1.**
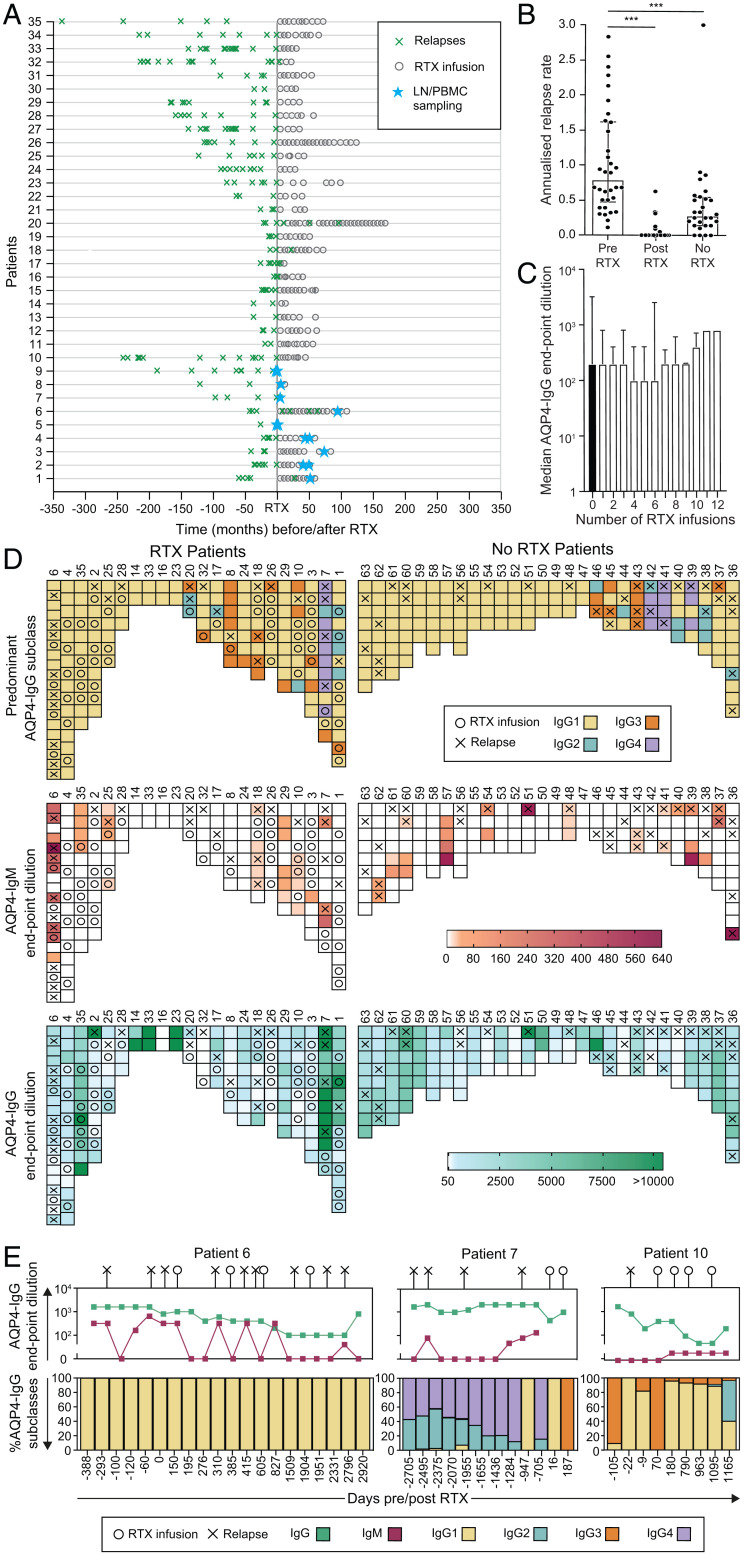
Serological associations with relapses and RTX administration in patients with NMOSD. (*A*) The effect of RTX administration on relapses (green crosses) in 35 NMOSD patients administered RTX at time 0. The timing of paired LN and PBMC sampling is shown (blue stars). (*B*) Mean ARR at last follow-up compared between patients before RTX and after RTX (*P* < 0.001) and to those administered no RTX (*P* < 0.001, Mann–Whitney *U* tests). (*C*) AQP4-IgG serum endpoint dilutions (shown as reciprocal of 1:dilution) were not reduced after multiple infusions of RTX (*P* = 0.99, Kruskal–Wallis test). (*D*) Heat maps to represent associations between relapses (X) and the dominant AQP4-IgG subclass (>50% of total AQP4-IgG, *Top*; determined by flow cytometry), AQP4-IgM (*Middle*; levels in red; determined by end-point titrations using live cell–based assays), and total AQP4-IgG (*Bottom*; levels in green; by endpoint titrations using live cell–based assays) in patients either administered RTX (*n* = 22) or naive to RTX (*n* = 28). (*E*) Longitudinal data in individual NMOSD patients (6, 7, 10), with negative values indicating days before the first RTX infusion; ***P < 0.001.

RTX administration in 35 of 63 NMOSD patients (median of seven infusions per patient, range of 1 to 14) was associated with a significant reduction in the ARR (median ARR of 0.79 to 0, *P* < 0.001, Wilcoxon signed-rank test; Fig. 1 *A* and *B*), which was apparent within a few months, and with the successful discontinuation of other immunotherapies in 31/35 patients (89%).

As reported elsewhere, longitudinal RTX infusions (median follow-up of 50 mo, range of 20 to 135) were not associated with significant reductions in serum AQP4-IgG levels (Fig. 1*C*) (19, 27).

**Fig. 2.**
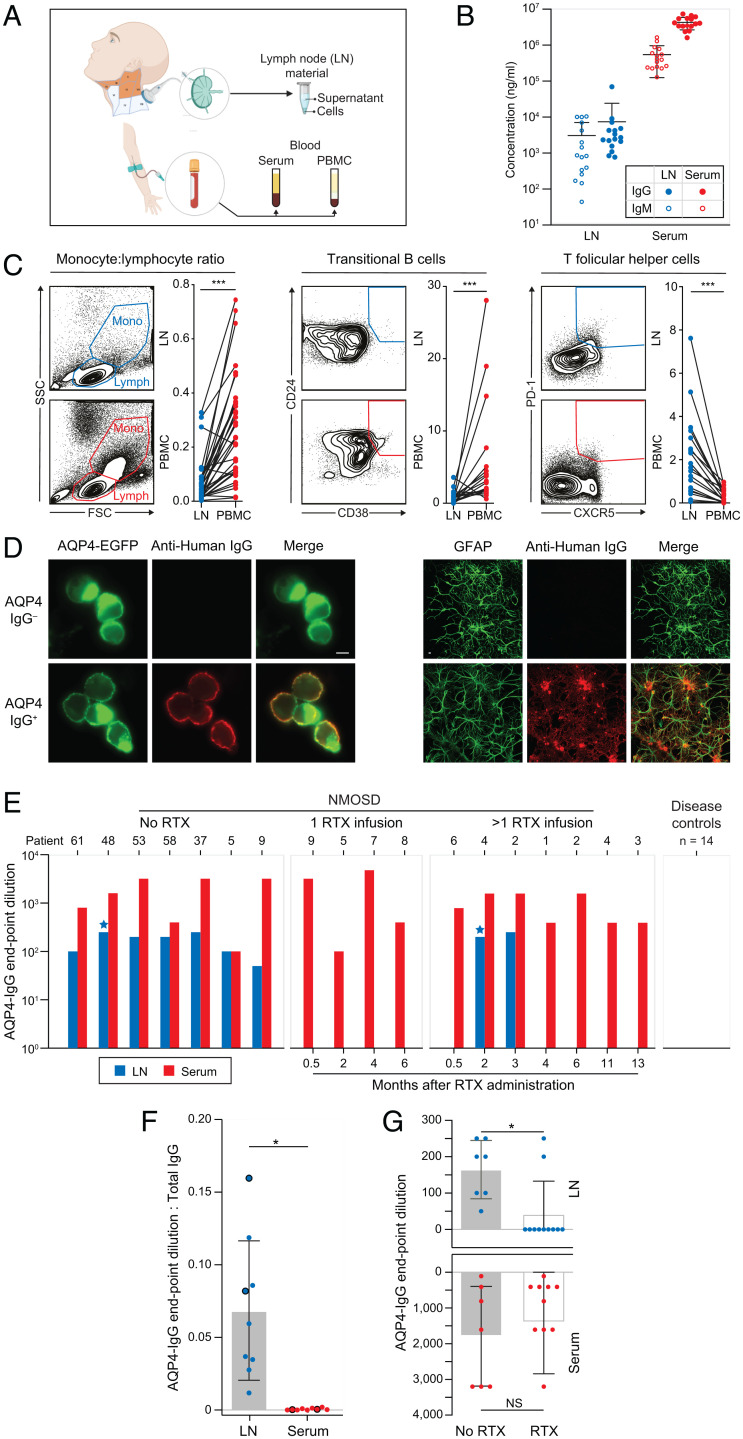
CLN aspirations contain AQP4 antibodies, which are abrogated after RTX administration. (*A*) CLNs across anatomical levels I, II, III, and V (orange) were aspirated under ultrasound guidance. Paired blood was obtained in parallel, resulting in cellular and soluble fractions from both sampled sites. (*B*) Markedly different levels of total IgG (filled symbols) and IgM (open symbols) were measured in LN aspirates (blue; IgG diluted at 1:800; IgM diluted at 1:100) versus matched sera of NMOSD patients (red; IgG diluted at 1:100,000; IgM diluted at 1:6,400). (*C*) Differences between PBMCs and LN cell populations are highlighted by the ratio of monocytes to lymphocytes (SSC = side scatter; FSC = forward scatter) and frequencies of both transitional B cells and Tfh cells (all *P* < 0.001, Wilcoxon signed-rank test), confirming that ultrasound LN sampling resulted in limited blood contamination of the LN aspirates. (*D*) AQP4-IgG was detected in LN aspirates by binding (anti-human IgG, red) to the surface of live AQP4–EGFP-transfected HEK293T cells (green; *Right*) and to the surface of live rat astrocytes (identified with GFAP, green; *Left*). (Scale bar, 10 µm). (*E*) AQP4-IgG levels in serum (red) and LN aspirates (blue) were measured in seven patients naive to RTX (of whom patients 5 and 9 went onto receive RTX), in four patients between 0.5 and 6 mo after one RTX infusion, and in seven patients between 0.5 and 13 mo after more than one RTX infusion (including two sampled longitudinally: patients 2 and 4). Blue stars indicate AQP4-IgM detected in two LN samples. (*F*) The ratios of AQP4-IgG levels (endpoint dilutions) to total IgG levels were compared between LN aspirates and sera (*P* = 0.02, Wilcoxon signed-rank test). Large dots highlight the two RTX-treated patients with detectable AQP4-IgGs from LN aspirates. (*G*) AQP4-IgG levels from LN aspirates (*P* = 0.04, Mann–Whitney *U* test) and serum (nonsignificant) in patients who did versus did not receive RTX; NS, not significant; *P < 0.05; **P < 0.01; ***P < 0.001.

**Fig. 3.**
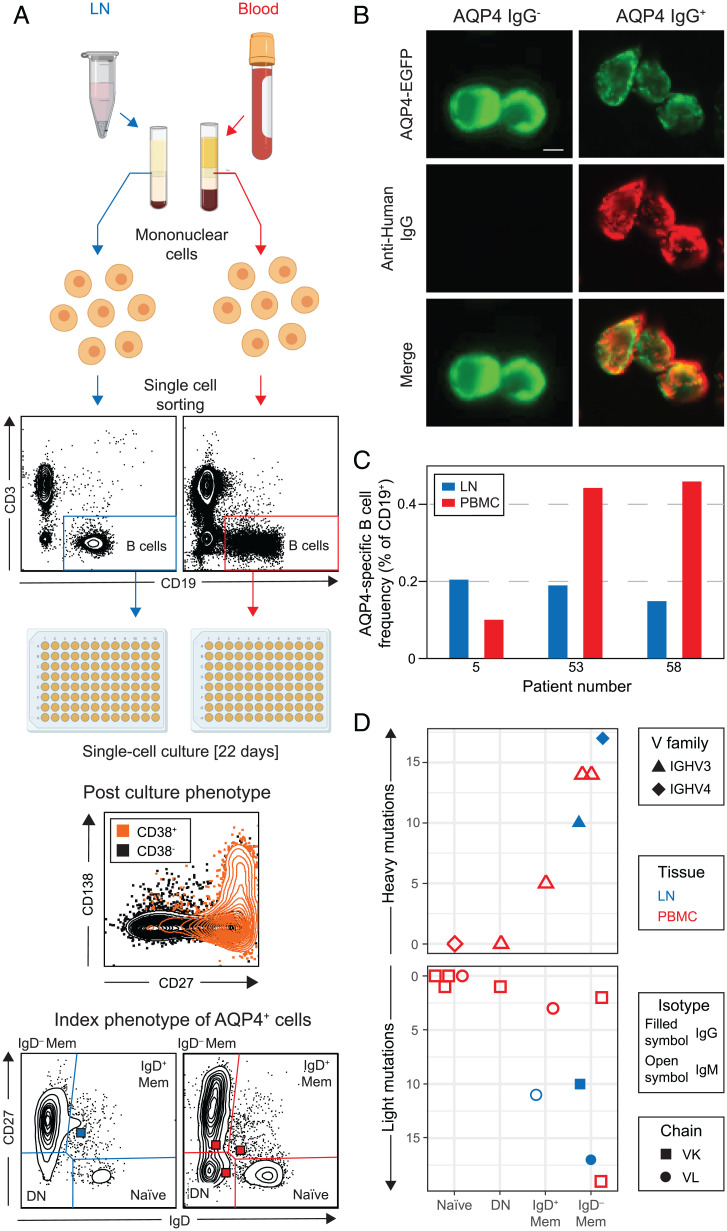
AQP4-specific B cells characterized from LNs and blood of patients with NMOSD. (*A*) Single B cells (CD3^−^CD19^+^) from paired blood (red) and LN (blue) samples were labeled with detection antibodies, index sorted as single cells, and cultured. By day 22, ∼50% of cells proliferate and differentiate into antibody-secreting cells (CD19 cells gated to show CD27, CD38, and CD138 expression). Indexing revealed the original B cell subsets: naive, double-negative (DN), IgD^−^, and IgD^+^ memory (Mem). (*B*) AQP4-reactive IgGs/IgMs in culture supernatants were identified by reactivity (red) directed against live HEK293T cells, which expressed surface AQP4–EGFP (green). (Scale bar, 10 µm.) (*C*) AQP4-specific B cell frequencies detected from three patients in LNs (blue) and PBMCs (red). (*D*) The heavy and light chains of these AQP4-specific BCRs (7 and 11 recovered, respectively) arose from all four studied B cell subsets and showed varied gene families (heavy variable IGHV3 as triangles and IGHV4 as diamonds) and light chain usage (kappa or lambda as squares or circles, respectively) from both LNs (blue) and PBMCs (red). The AQP4-reactive isotype detected in supernatants is shown using open (AQP4-IgM) or filled (AQP4-IgG) symbols; DN, double-negative cells; IGHV, immunoglobulin heavy chain variable region.

To study proxy markers of GC activity in peripheral blood, AQP4-IgG subclasses and AQP4-IgM levels were measured from 406 longitudinal serum samples collected over a median of 41 mo (range of 2 to 145 mo; Fig. 1 *D* and *E* and *SI Appendix*, Fig. 2). Ten of 22 (45%) RTX-treated patients and 17 of 28 (61%) patients treated with other immunotherapies showed an exclusive IgG1 predominance of AQP4-IgG throughout their disease course, followed, in descending frequency, by AQP4-reactive IgG3, IgG2, and IgG4. A change in the dominant AQP4-IgG subclass was noted around the time of 24/61 (39%) clinical relapses but only at 40/255 (16%) timepoints remote (>8 wk) from a relapse (odds ratio [OR] of 3.49 [range of 1.89 to 6.29], *P* = 0.0001; Fisher’s exact test). This shift in the predominant AQP4-IgG subclass may reflect active class-switch recombination, a surrogate of GC activity. If this was driven by de novo entry of naive B cells into GC reactions, AQP4-IgMs may also be generated. Indeed, across multiple timepoints, serum AQP4-IgM was detected in 22/50 (44%) patients, preferentially in association with 29/61 (48%) relapses versus only 37/316 (12%) samples obtained remote from relapses (*P* < 0.0001, OR of 6.83 [range of 3.64 to 12.26], Fisher’s exact test; Fig. 1 *D* and *E* and *SI Appendix*, Fig. 3). Overall, the presence of either AQP4-IgM or a shift from the AQP4-IgG1 subclass was associated with relapses (43/61 versus 73/255; OR of 6.0 [range of 3.3 to 10.8], *P* < 0.0001). Taken together, these findings implicate GC activity as a source of AQP4 antibody production around the time of relapses.

### Local Synthesis of AQP4-IgG in dCLNs Is Abrogated by RTX.

To test this hypothesis directly in humans, 36 dCLN aspirations were performed in 14 patients with NMOSDs (*n* = 18 aspirations) and in 14 disease controls (*n* = 18 aspirations; Fig. 2*A* and *SI Appendix*, Table 2). In dCLNs, total IgG and IgM levels were ∼1,500- and ∼500-fold lower than in sera (Fig. 2*B*), and limited contamination by blood products was confirmed based on the markedly different leukocyte populations observed from within these two compartments (Fig. 2*C*). In comparison to blood, dCLNs showed lower proportions of monocytes (monocyte:lymphocyte ratio; median of 0.21 [range of 0.01 to 0.70] in blood and 0.03 [range of 0.003 to 0.23] in dCLNs; 6.6-fold reduction; *P* < 0.0001) and transitional B cells (CD19^+^CD20^+^CD24^+^CD38^+^; median of 2.8 [range of 0.6 to 28] in blood and 0.72 [range of 0 to 3.6] in dCLNs; 3.9-fold reduction; *P* < 0.0001) but higher frequencies of Tfh cells (CD3^+^CD4^+^PD-1^+^CXCR5^+^, median 0.19 [range of 0 to 0.96] in blood and 1.64 [range of 0.07 to 7.6] in dCLNs; 8.6-fold increase; *P* < 0.0001; all Wilcoxon signed-rank test) (28).

**Fig. 4.**
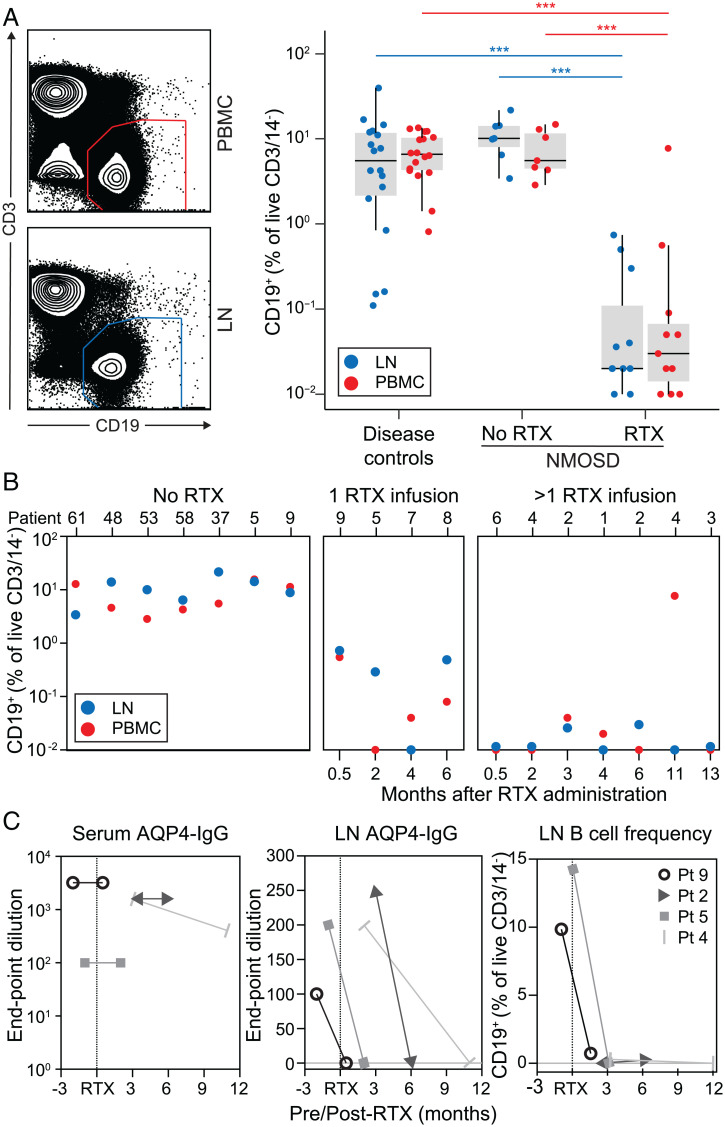
Intranodal B cells are rapidly depleted after RTX administration. (*A*) After RTX administration, B cells (CD19^+^ of CD14^−^DAPI^−^CD3^−^) were markedly depleted from both LNs (blue, *P* < 0.001, Mann–Whitney *U* test) and blood (red, *P* < 0.001, Mann–Whitney *U* test) in comparison to both disease controls and NMOSD patients that did not receive RTX. (*B*) Overall, the LN depletion was more pronounced in patients after more than one versus only one RTX infusion (*P* = 0.04, Mann–Whitney *U* test). (*C*) From two patients sampled before and after the first RTX infusion (patients 5 and 9) and two sampled at sequential timepoints after RTX infusion (patients 2 and 4), the unchanged serum AQP4-IgG levels (red) contrast with marked reductions in LN aspirates of both AQP4-IgG (blue) and LN B cell frequency (black); ***P < 0.001.

No AQP4-IgGs were detected in dCLN aspirates or sera from 14 disease controls. By contrast, all seven dCLN aspirates from RTX-naive NMOSD patients showed AQP4-IgG, as detected with IgG binding to the extracellular domain of HEK293T-expressed AQP4 (endpoint dilutions of 0 to 1:250; median of 50) and to the surface of live astrocytes (Fig. 2 *D* and *E*). In these seven patients, serum AQP4-IgG levels ranged from endpoint dilutions of 1:100 to 1:3,200 (median of 1:400). The ratio of AQP4-IgG to total IgG was ∼200-fold higher (median of 0.07, range of 0.02 to 0.12) in dCLN aspirations versus matched sera (median of 0.0003, range of 0 to 0.002; *P* = 0.016; Wilcoxon signed-rank test; Fig. 2 *E*–*G*), suggesting local (“intranodal”) synthesis of AQP4-IgG. This concept was supported by the detection of AQP4-IgMs in two dCLN aspirates but not in matched sera (Fig. 2*E* and *SI Appendix*, Fig. 4). By contrast, in dCLN aspirates sampled at varied timepoints after RTX administration, AQP4-IgG or AQP4-IgM was detected in only 2/11 patients (18%; *P* = 0.002, Fisher’s exact test; Fig. 2*G*). These collective observations indicate that intranodal synthesis of AQP4-IgG is effectively abrogated by RTX administration. By extension, these results suggest that AQP4-specific B cells exist within NMOSD patient dCLNs before RTX administration.

### AQP4-Specific B Cells in LNs and Blood.

To identify these AQP4-specific B cells, single B cells from both dCLNs and blood of RTX-naive NMOSD patients were index sorted and individually exposed to cytokines, which induced proliferation and differentiation into clonal antibody–secreting cells (CD19^+^CD27^++^CD38^++^ and often CD138^+^; Fig. 3*A*). After 22 d in culture, supernatants were assessed for AQP4-IgM and IgG reactivities. Across three patients, 11 of 8,293 cultured B cells secreted either AQP4-IgM or IgG (Fig. 3*B*), equating to ∼0.2% of CD19^+^ B cells from blood and dCLNs after accounting for the ∼50% in vitro survival rate of these cultures (Fig. 3*C*). Cell indexing revealed that these AQP4-specific B cells were derived from naive (*n* = 4), double-negative (*n* = 1), IgD^+^ memory (*n* = 2), and IgD^−^ memory (*n* = 4) populations (Fig. 3 *A* and *D* and *SI Appendix*, Table 3 and Fig. S5). All three AQP4 antibodies from the dCLNs were derived from the switched memory (IgD^−^) population and included the two AQP4 reactivities detected as IgGs in supernatants. All others were detected as AQP4-IgMs. Cognate paired heavy and light chains from naive B cells were unmutated with increasing mutation loads observed through double-negative, IgD^+^ memory, and IgD^−^ memory cells (Fig. 3*D*). All showed highly variable VDJ gene usage and with ∼2:1 kappa:lambda chain expression (*SI Appendix*, Table 3). In summary, AQP4-specific B cells were isolated from both peripheral blood mononuclear cells (PBMCs) and dCLNs and arose from genetically diverse lineages within multiple B cell subsets from early unmutated and later stages of B cell development.

### Effective Depletion of dCLN B Cells after RTX.

From both blood and dCLNs, RTX administration was associated with 198- and 430-fold median reductions in respective CD19^+^ B cell frequencies (*P* < 0.001, Mann–Whitney *U* test, Fig. 4*A*), without significant alterations in the relative frequencies of B cell subsets, antibody-secreting cells, or Tfh cells and only a modest drop in CD20^+^ T cell frequencies (*SI Appendix*, Fig. 6). The intranodal B cell depletion persisted for several months from RTX dosing and was most marked in the NMOSD patients who had received >1 RTX infusion (median of 3 dCLN B cells, range of 0 to 56) by comparison to either those who had been administered a single infusion (median of 49 dCLN B cells, range of 9 to 230; *P* = 0.04, Mann–Whitney *U* test) or the RTX-naive cohort (median of 6,954 dCLN B cells, range of 1,603 to 65,276; *P* < 0.0001, Mann–Whitney *U* test) (Fig. 4*B*). To validate these results longitudinally within individuals, AQP4-IgG levels and B cell frequencies were analyzed from four patients who underwent paired sampling of blood and the same dCLN on two occasions, before and after RTX administration (*n* = 2) and following RTX administration (*n* = 2; Fig. 4*C*). In all cases, over 3 to 12 mo from RTX administration, the serum AQP4-IgG levels were unchanged, whereas AQP4-IgG levels in dCLNs fell from endpoint dilutions of 1:100 to 1:250 to undetectable, and the dCLN B cell populations became (*n* = 2) or remained undetectable (*n* = 2). Hence, RTX robustly depleted B cells and AQP4-IgGs in dCLNs without changes in serum AQP4-IgGs.

## Discussion

Our approach of sampling dCLNs in patients with NMOSDs provides direct evidence for the generation of AQP4-IgGs in human GCs. The observed intranodal AQP4-specific B cells and intranodal synthesis of AQP4-IgGs are complemented by indirect measures of GC activity from serum, including a switch in AQP4-IgG subclasses and spikes in AQP4-IgM, which significantly associated with clinical relapses. Collectively, these direct and proxy measures implicate longitudinal GC activity as a potential driver of AQP4-IgG production and provide useful predictors of relapses in patients with NMOSDs (29). Of immediate therapeutic relevance, sampling dCLNs before and after RTX administration showed that this anti-CD20 therapy markedly abrogates both intranodal B cells and the corresponding intranodal AQP4-specific GC activity. This direct effect on LNs may disrupt actively maturing GCs in NMOSDs despite maintained serum AQP4-IgG levels, simultaneously accounting for its rapid clinical efficacy and providing a potential explanation for the observed clinical–serological paradox. More generally, our study presents an ex vivo human paradigm to determine multiple biomarkers of GC activity as valuable direct and indirect measures for clinical trials and as a platform to better understand both the biology of other autoimmune conditions and the mechanisms of both GC-directed immunotherapeutics and vaccinations (30, 31).

Our observations, alongside available studies, collectively identify many elements of a classical antigen-specific GC reaction in patients with NMOSDs. First, serum AQP4-IgG levels are ∼1,000-fold higher than in cerebrospinal fluid (32), supporting peripheral initiation of AQP4-directed autoimmunization (9). Second, an early loss of peripheral B cell tolerance is consistent with our definitive isolation of AQP4-specific unmutated, germline IgM^+^ naive B cells from NMOSD patient PBMCs (12, 33) and with the observed clonal relationships between intrathecal AQP4-specific cells and peripheral double-negative and naive B cells (17). Although affinity was not measured directly in this study, the unmutated naive BCRs are likely to have a low affinity for AQP4 and may be the first lymphocytes to bind conformational AQP4 epitopes in the initiation of GC reactions. Thereafter, during clinical relapses, the observed spikes in AQP4-IgM suggest that GC activity is driven by the preferential recruitment of naive B cells rather than IgG^+^ memory B cell reactivation, a concept consistent with predominant naive B cell entry into GCs during antigen rechallenge animal models (34). Within GCs, it is likely that AQP4-specific T cells (35, 36) help naive AQP4-specific B cells to differentiate and mutate into higher-affinity AQP4-specific memory B cells, which we captured from both blood and dCLNs. The observed ∼0.2% frequency of AQP4-specific B cells is several-fold greater than most chronic antimicrobial responses (37) and implies that ongoing AQP4-directed autoimmunizations are actively occupying GCs in NMOSD patients. The slightly higher AQP4-reactive B cell frequency observed in blood versus dCLNs may reflect well-established difficulties in culturing GC B cells (10), and, moving forward, our data can be used to formally power future studies to evaluate these parameters.

The effect of RTX on LN B cell depletion has yielded conflicting results to date, which are not easily reconciled by considering factors such as the use of animal versus human models, the anatomical location of sampled LNs, the duration between RTX administration and sampling, or the underlying clinical condition and therapies (24, 25, 38–41). Here, by longitudinally sampling dCLNs in a well-defined patient population, our data show a ∼400-fold depletion of dCLN B cells after a single course of RTX, an effect size that suggests a major therapeutic contribution. Without a focused time course of blood versus dCLN dynamics, it remains possible that peripheral depletion is the dominant effect, followed by limited migration of B cells into LNs. Nevertheless, in humans, our data show that B cell retention in LNs is unlikely to be a valid explanation for RTX resistance. While other LN basins have not been explored, we propose dCLNs as the most plausible site for GC initiation in a disease driven by a CNS-predominant antigen, as they directly drain CNS lymphatics (26). In light of our observations, AQP4 antigen detection from human dCLN-based GCs is a realistic future aim, especially given that blood contamination in aspirates appeared minimal. Yet, histological features of ectopic GCs have been described within the orbits of two patients with NMOSDs (42), and it may be that CNS AQP4-rich sites are seeded with AQP4-specific B cells after their migration into the CNS (16). An additional limitation of our study is the sampling from NMOSD patients with varied other immunotherapy regimens and sampling during both relapses and remission. Future studies should aim to independently characterize the immunobiology underlying these disease phases.

The residual AQP4-IgG observed after RTX administration is likely to arise from bone marrow-niched LLPCs, traditionally proposed to account for the medium- to long-term production of several antibody specificities (14, 43). However, given the typically marked clinical efficacy of RTX in NMOSDs, we propose a parsimonious theory to explain this well-recognized clinical–serological paradox (8, 21). AQP4-IgG levels have been shown to gradually rise over several years until clinical features of NMOSDs manifest (44). These kinetics may be consistent with a gradual ongoing affinity maturation in GCs. Over time, some of the low- to moderate-affinity AQP4-specific B cells from GCs may acquire bone marrow niches as LLPCs. Given their intrinsically high IgG secretion rates, these LLPCs then account for the majority of circulating AQP4-IgG. Yet, if the most pathogenic AQP4-IgG species (5) continue to affinity mature further in GCs and their appearance is coincident with the onset of NMOSD symptoms, RTX may act to effectively deplete the GC B cells most likely to initiate disease and yet only minimally reduce total AQP4-IgG levels. This model may also explain why reconstitution of circulating memory B cells after RTX appears to be a biomarker of incipient relapses and a clinical indication for repeat RTX administration (45). If this model is accurate and the CD19^+^ LLPC population secretes AQP4-IgG with low pathogenic potential, the recently approved anti-CD19 therapies (46) may offer limited incremental benefit over anti-CD20 therapies, and their effective relapse prevention may be better explained by the depletion of CD19^+^CD20^+^ B cells. Direct bone marrow access may be required to accurately compare AQP4-IgGs from LLPCs to those within dCLNs.

In conclusion, our study highlights a paradigm to study an autoantigen-specific response in human secondary lymphoid organs, and our data form the basis of a hypothesis to explain the clinical efficacy of RTX across autoantibody-mediated conditions despite the lack of CD20 expression on LLPCs. Our findings have implications for monitoring patients and evaluating the basis of treatment escalation and aim to directly appreciate the underlying disease biology. Further, biomarkers of GC activity are likely to have broad applicability in the clinical arena and may be an avenue toward precision medicine across autoantibody-mediated conditions.

## Materials and Methods

### Participants.

Sixty-three patients with NMOSDs and serum AQP4-IgG (7) were seen as part of routine clinical practice in expert NMOSD clinics (Oxford/Liverpool, UK) and were selected based on availability of longitudinal serum samples archived at −80 °C. Thirty-five of 63 patients had received RTX that was initially administered as 1g intravenously twice separated by a fortnight, followed by 1g maintenance interval doses prompted by the return of detectable circulating CD19^+^ counts. Information collected retrospectively from the case notes included demographics, clinical features, and timings of medication administration and relapses, which were used to calculate an ARR (*SI Appendix*, Tables 1 and 2). The full study protocol was approved by Yorkshire/Humber and South-Central UK research ethical committees (REC16/YH/0013, REC16/SC/0224, and REC14/SC/005). Written informed consent was obtained from all participants, including 14 disease controls (with autoimmune encephalitis, *n* = 11, or migraine, *n* = 3; select controls also in ref. 47). For patients and controls, demographics, past and current immunotherapies, and sampling times are detailed in *SI Appendix*, Tables 1 and 2.

### Fine Needle Aspiration (FNA) Procedure.

Ultrasound guidance was used to locate dCLNs (at anatomical levels I, II III, or V; *SI Appendix*, Table 2), which were accessed under visualization with a 23-gauge needle. After two passes, aspirates were diluted in phosphate-buffered saline (to 1:50) and centrifuged to separate cellular and soluble fractions. The cellular fraction contained a median of 1.5 × 10^6^ live cells per FNA (range of 2.4 × 10^5^ to 3.5 × 10^6^). From the soluble fraction, total IgG/IgM and AQP4-IgG/IgM were determined. The overall breakdown of serum and dCLN assays is shown in *SI Appendix*, Fig. 1.

### Antibody Detection Methods.

From 63 patients, a total of 406 serum samples were tested for AQP4-IgGs and AQP4-IgMs using a live cell–based assay (48). Briefly, HEK293T cells were transiently transfected to express surface AQP4 (using the M23 isoform C-terminally fused to enhanced green fluorescent protein [EGFP]) and, while live, incubated with either patient serum (starting dilution of 1:20) or the supernatant fraction of dCLN aspirates (starting dilution of 1:50) before fixation and washing. Subsequently, binding was visualized with Alexa Fluor 594–conjugated secondary antibodies targeting the Fc regions of IgM (A21216, Invitrogen) or IgG (709-585-098, Jackson Labs). All samples were titrated to endpoint dilution, and, before AQP4-IgM determination, protein G Sepharose beads (17-0618-01, GE) were used to deplete IgG to prevent the potential for IgG–IgM cross-competition in unfractionated sera. To quantify total AQP4-IgG levels and subclasses, all 319/406 serum samples (from 50/63 patients) with an endpoint dilution of >1:20 (1:50 to 1:40,000) were used to label transiently transfected AQP4–EGFP-expressing HEK293T cells in suspension. After washing and fixation, in parallel assays using an Attune NxT flow cytometer, the IgGs bound to live cells (DAPI^−^) were detected with subclass-specific antibodies (IgG1 hinge–Alexa Fluor 647 [9052-31], IgG2 Fc–Alexa Fluor 647 [9070-31], IgG3 hinge–Alexa Fluor 647 [9210-31], IgG4 Fc–phycoerythrin [PE; 9200-09], Southern Biotech). As described previously for other autoantibodies (49), total and subclass levels of AQP4-IgG were calculated by the delta median fluorescence intensity of the transfected (single cells/DAPI^−^EGFP^+^ gates) minus untransfected (single cells/DAPI^−^EGFP^−^ gates) cells, and normalized antibody-binding capacities were calculated with calibration beads (Quantum Simply Cellular microspheres, Bangs Laboratories). The cutoff for positivity was determined for each subclass using 10 healthy control serum samples (mean value plus three SDs), and the dominant AQP4-IgG subclass was defined as comprising >50% of total AQP4-IgG. Samples taken after 8 wk of a relapse were characterized as “remote”.

Total IgG and total IgM from supernatants and sera were measured by enzyme-linked immunosorbent assay (Bethyl Laboratories).

### Primary Mixed Cultures of Astrocytes and Neurons.

Mixed neuronal–astrocyte cultures were prepared from rat hippocampi at embryonic day 18, as described previously (50). Briefly, hippocampi were digested in trypsin, mechanically dissociated, and plated with neurobasal medium/B27 supplement (1:50; Thermo Fisher). After 21 to 28 d in vitro, patient sera (1:100) or the soluble fraction of dCLN aspirates (1:50) was diluted in conditioned medium and incubated with the live-cell culture preparation for 30 min at 37 °C and fixed with 4% paraformaldehyde. To visualize bound human antibodies, either a goat anti-human IgG or IgM Fc region, cross-absorbed, unconjugated secondary antibody was applied (Thermo Fisher; 1:750), followed by a fluorescently conjugated tertiary antibody (donkey anti-goat IgG, Alexa Fluor 568; A-11057, Thermo Fisher). To identify astrocytes, cells were permeabilized (0.1% Triton X-100) and incubated with a commercial antibody against glial fibrillary acidic protein (GFAP; DAKO, Z0334; 1:2,000), which was detected with a goat anti-rabbit IgG secondary antibody (Alexa Fluor 488; A-11008, Thermo Fisher; 1:750).

### Flow Cytometry and Cell Sorting.

Fresh dCLN mononuclear cells and matched PBMCs were isolated on a Ficoll density gradient. Cells were incubated with normal mouse serum to block nonspecific binding, and surface phenotypes were determined with the following commercial fluorochrome-conjugated antibodies: CD45 (HI30, AF700, BioLegend), CD3 (UCHT1, Pacific Blue, BioLegend), CD14 (HCD14, Pacific Blue, BioLegend), CD19 (SJ25C1, APC-Cy7, BD Biosciences), CD20 (2H7, BV711, BioLegend), CD24 (ML5, BV510, BioLegend), CD27 (O323, BV605, BioLegend), CD38 (HIT2, PE, BD Biosciences), IgD (IA6-2, fluorescein isothiocyanate, BD Biosciences), CD4 (RPA-T4, PE-CF594, BD Biosciences), CXCR5 (J252D4, PE-Cy7, BioLegend), and PD-1 (RMP1-30, APC, BioLegend). Subsequently, cells were washed, and DAPI was added before analysis with an Attune NxT flow cytometer. Flow cytometric data were manually gated using FlowJo software (TreeStar Inc; *SI Appendix*, Fig. 1 *B* and *C*).

### Single B Cell Cultures and Immunoglobulin Chain Retrieval.

A FACSAria III was used to index sort single B cells prelabeled with antibodies against CD19, IgD, and CD27 into individual wells of 96-well plates, where they were cultured with MS40L-low feeder cells (a kind gift from Dr. G. Kelsoe) in Roswell Park Memorial Institute (RPMI) 1640 containing 10% fetal bovine serum (Thermo Scientific), recombinant human interleukin-2 (100 µg/mL, Peprotech), and B-cell-activating factor (100 µg/mL, Peprotech) at 37 °C in 5% CO_2_. On day 22, culture supernatants were harvested for AQP4-IgG and AQP4-IgM detection. From wells with detectable AQP4-IgM or AQP4-IgG, transcripts were preserved (RNAsin, Promega), and, after reverse transcription, heavy- and light-chain PCRs were performed, as previously described (51). AQP4-specific immunoglobulin variable region sequences were identified and analyzed using https://www.ncbi.nlm.nih.gov/igblast and https://www.imgt.org.

### Statistical Analysis.

GraphPad Prism (v8; GraphPad Software Inc.), R (52), and Adobe Illustrator were used for statistical analysis and data presentation.

## Supplementary Material

Supplementary File

## Data Availability

All study data are depositied at https://github.com/jtheorell/Damato_NMO_RTX; many included in the article and/or *SI Appendix*.
