# Supplementary material for: Rituximab abrogates aquaporin-4–specific germinal center activity in patients with neuromyelitis optica spectrum disorders
Source: Proc Natl Acad Sci U S A. 2022 Jun 6;119(24):e2121804119. doi: 10.1073/pnas.2121804119 (PMC9214492; doi:10.1073/pnas.2121804119)
Supplement: Supplementary File [file pnas.2121804119.sapp.pdf]

Supplementary information

Rituximab abrogates aquaporin-4-specific germinal centre activity in patients with Neuromyelitis optica spectrum disorders

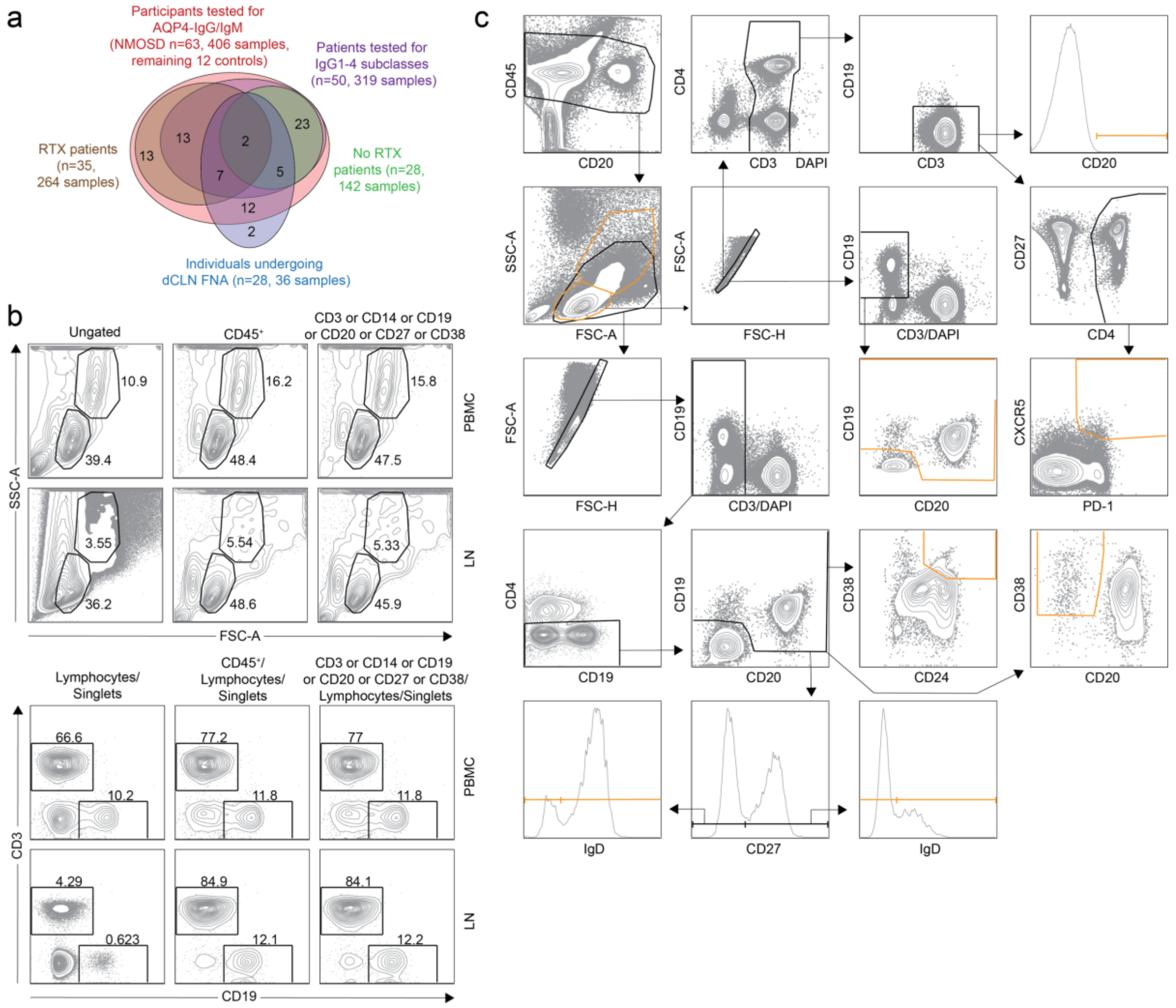

Supplementary Figure 1. Summary of sampling and testing procedures and flow cytometry gating strategies. **a**. Venn diagram to show how the samples were distributed among patients with and without RTX administration and disease controls. The two patients in the middle green field represent individuals for which assessments, including dCLN aspiration, was performed both before and after the first RTX dose. The two individuals solely in the outer blue circle represent 2/14 disease controls who underwent dCLN aspirations. **b**. Gating strategies. CD45 staining was available for 23 of 36 dCLN/PBMC sample pairs. When not included in the panel, events were accepted if positive for any of CD3, CD14, CD19, CD20, CD27 or CD38 (‘Boolean gate’). Representative example showing the phenotype of the CD45 positive population or events within the Boolean gate

were indistinguishable. **c**. Overarching gating strategy for the flow cytometry data analysis with red gates representing populations that were analysed within the study. Grey gates illustrate populations used to derive red gates. AQP4= aquaporin 4; dCLN= lymph node; FNA= fine needle aspiration; IgG = Immunoglobulin G; IgM= Immunoglobulin M; PBMC= peripheral blood mononuclear cells; RTX = Rituximab.

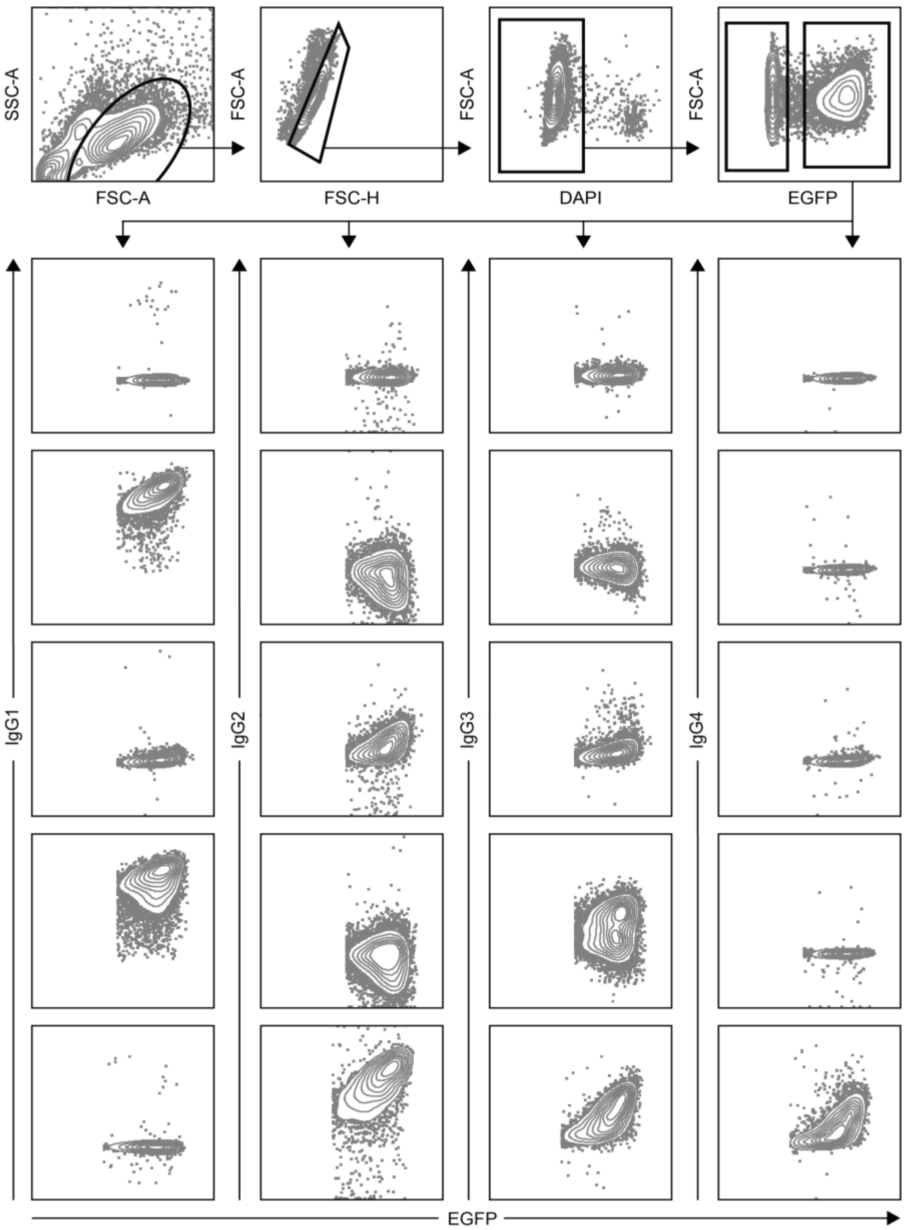

Supplementary Figure 2. Detection of AQP4-IgG subclasses. Flow cytometry gating and representative examples of AQP4-IgG1-4 subclasses detection in four NMOSD patients. AQP4= aquaporin 4; EGFP= enhanced green fluorescent protein; IgG = Immunoglobulin G; NMOSD = Neuromyelitis Optica spectrum disorders.

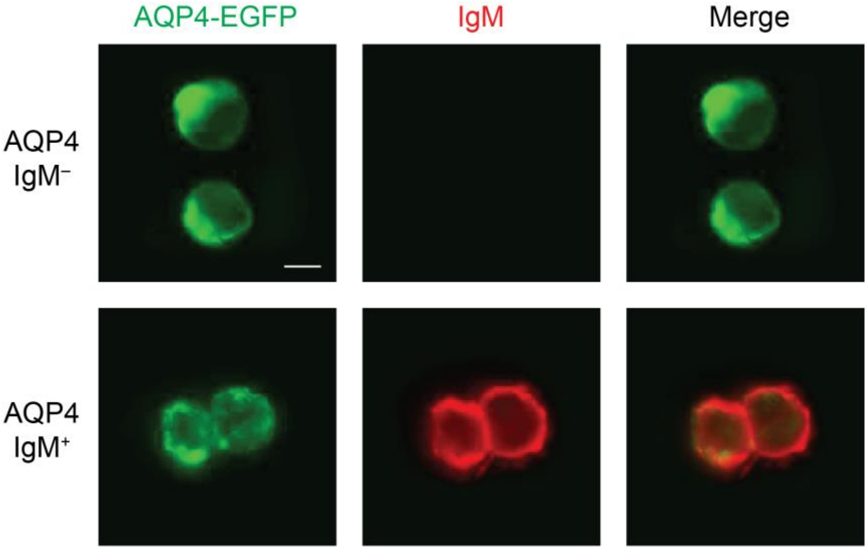

Supplementary Figure 3. Detection of AQP4-IgM by live cell-based assay. After depletion of IgG, AQP4-IgM were detected in serum by binding (anti-human IgM, red) to the surface of live AQP4- EGFP transfected HEK293T cells (green). AQP4= aquaporin 4; EGFP= enhanced green fluorescent protein; HEK= Human embryonic kidney; IgM = Immunoglobulin M.

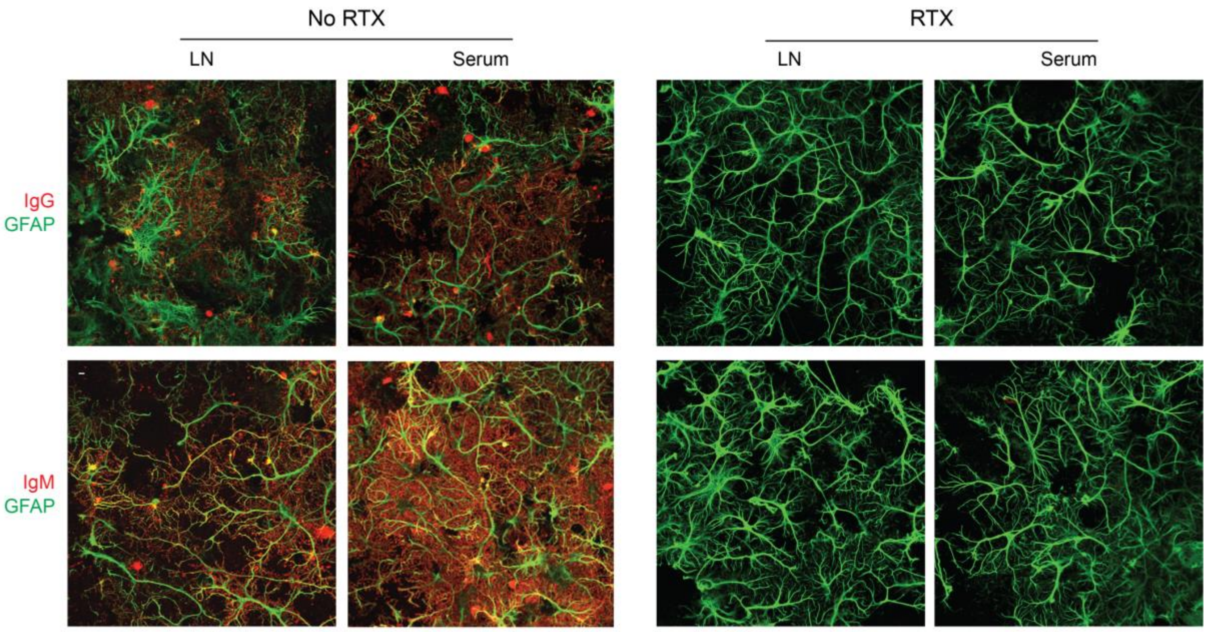

Supplementary Figure 4. RTX administration is associated with absence of detectable AQP4-IgG/M in dCLN aspirates and matched sera. Both matched sera and dCLN aspirates from seven RTX-naïve NMOSD patients contained detectable surface astrocyte binding (and AQP4-IgG, e.g. Figure 2D). In addition, 2/7 of these LN aspirates, but not the matched sera, contained AQP4-IgM (shown in Figure 2E). By contrast, astrocyte binding was only detected in 2/11 LN aspirates from patients administered RTX. A commercial antibody specific for GFAP confirmed their designation as astrocytes. Scale bar 10 μm. AQP4= aquaporin 4; dCLN = deep cervical lymph node; GFAP= glial fibrillary acidic protein; IgG = Immunoglobulin G; IgM= Immunoglobulin M; LN= lymph node; NMOSD = Neuromyelitis Optica spectrum disorders; RTX = Rituximab.

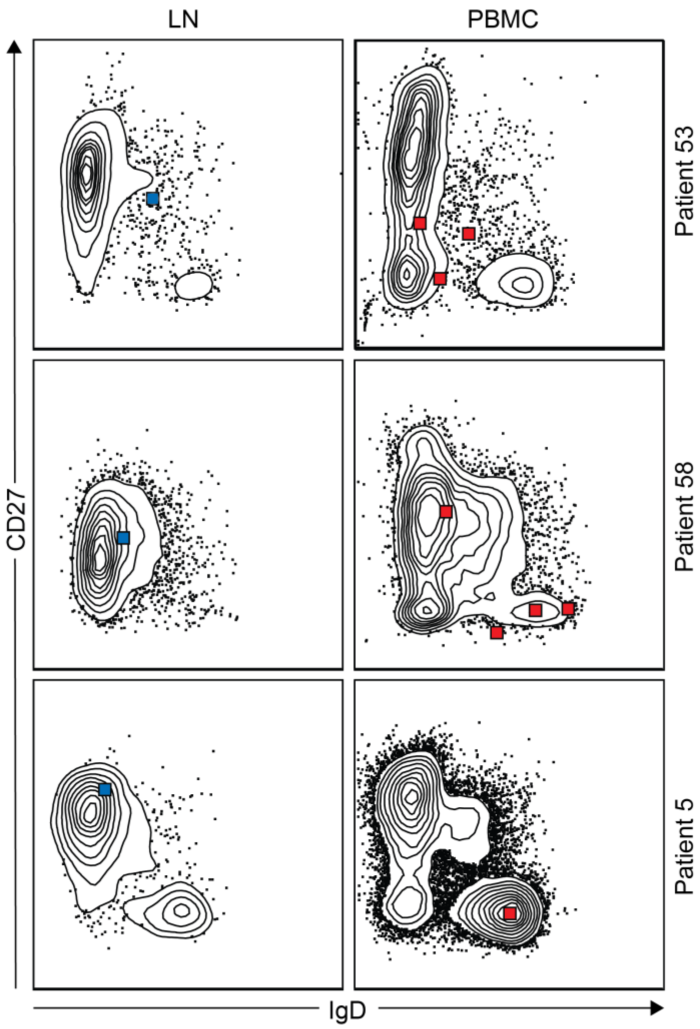

Supplementary Figure 5. Phenotypes of AQP4-expressing B cells. Red dots represent cells found in PBMC whereas blue dots represent cells found in dCLNs, across three patients after index sorting. AQP4= aquaporin 4; dCLN= lymph node; PBMC= peripheral blood mononuclear cells.

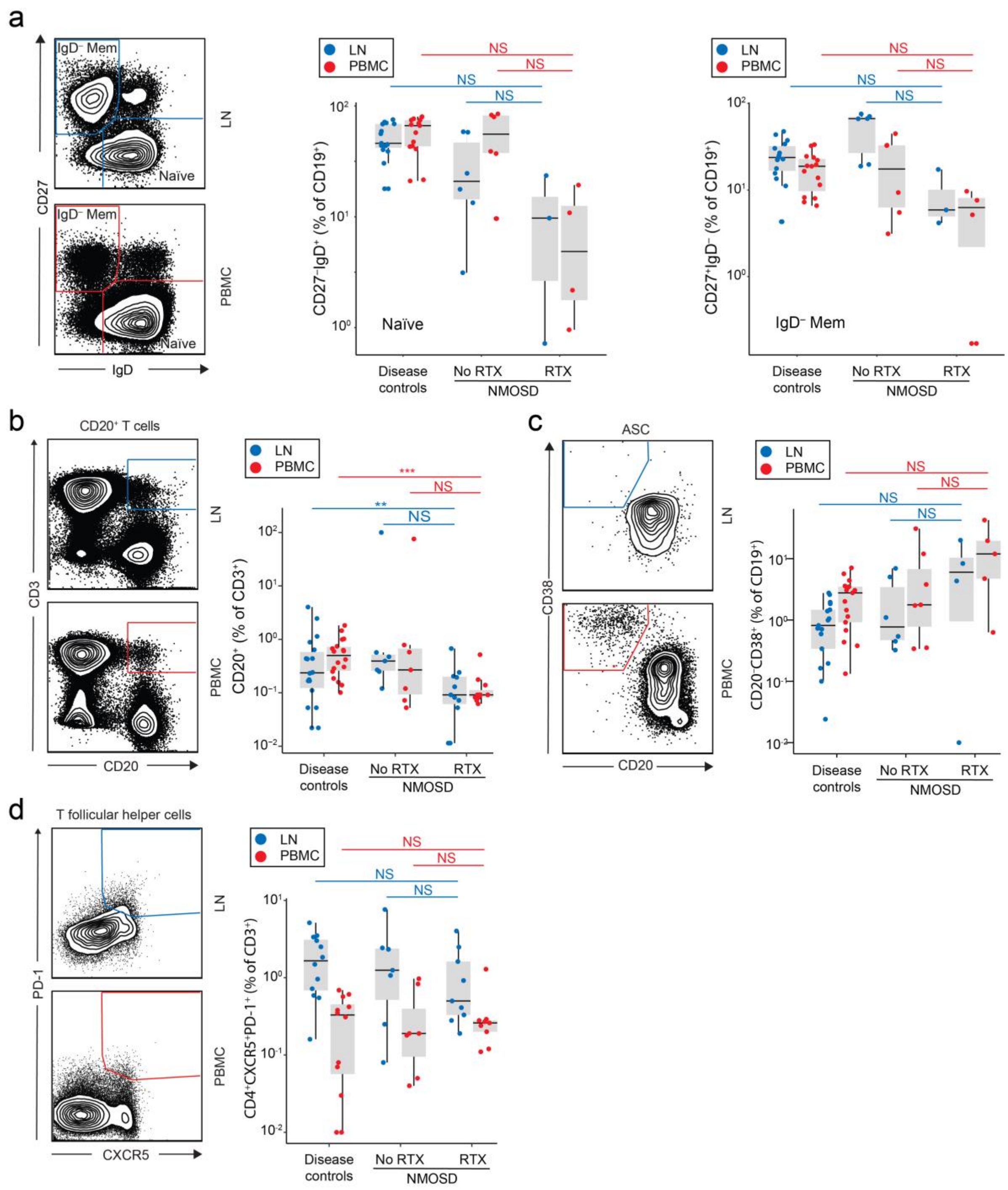

Supplementary Figure 6. B- and T-cell subset frequencies in PBMC and dCLNs compared across disease controls and NMOSD patients after or naïve to RTX. a. $IgD^{+}CD27^{-}$ naïve and $IgD^{–}CD27^{+}$ memory B cell subset frequency expressed as a percentage of total $CD19^{+}$ B-cells. **b.** $CD20^{+}CD3^{+}$ subset frequency ($CD20^{+}$ T cells) expressed as a percentage of total $CD3^{+}$ cells. **c.** $CD19^{+}CD20^{–}CD38^{+}$ antibody-secreting cell (ASC) subset

frequency expressed as a percentage of total CD19$^+$ B-cells. **d.** CD4$^+$CXCR5$^+$PD-1^{++} T follicular helper subset frequency expressed as a percentage of total CD3$^+$ cells. For all populations, only individuals with more than 50 cells in the parent population (i.e. CD19$^+$ or CD3$^+$) were included. A median of 6704 B cells events (range 61-99629) was analysed. Statistical analyses with Mann-Whitney U-test after Benjamini-Hochberg correction for the 20 overall comparisons. ASC = antibody-secreting cell; dCLN= lymph node; NMOSD = Neuromyelitis Optica spectrum disorders; PBMC= peripheral blood mononuclear cells; RTX = Rituximab; TfH= T follicular helper cells. NS= not significant; $^{**}p < 0.01$; $^{***}p < 0.001$.

	RTX (N=35)	No RTX (N=28)	
Female proportion	30/35 (86%)	25/28 (89%)	p=0.48°
Mean age at NMOSD onset	36	45	p=0.007‡
Mean disease duration (months)	132	128	p=0.90‡
Median ARR	0.89†	0.28	p<0.001°
Median number of immunotherapies	2†	1	p=0.10°
Median AQP4-IgG titre	200†	800	p=0.09°

Supplementary Table 1. Clinical and demographic characteristics of the two NMOSD cohorts included in the study. Patients data were tested for normality (Gauss distribution with the Anderson Darling, D'Agostino & Pearson, Shapiro-Wilk and Kolmogorov-Smirnov tests). Normally distributed data were analysed with an unpaired t-test (‡); otherwise, a Mann-Whitney U test (°) was employed. †Data prior to rituximab administration. AQP4= aquaporin 4; ARR = annualised relapse rate; IgG = Immunoglobulin G; NMOSD = Neuromyelitis Optica spectrum disorders; RTX = Rituximab.

Cohort	Disease (patient number)	Gender/Age at disease onset	Previous IT	ARR Pre-RTX	ARR Post-RTX	IT at time of FNA	Disease duration at FNA (months)	Number of RTX infusions prior to FNA	Time from last RTX at FNA (months)	Lymph node level for FNA
No RTX	NMOSD (#37)	F/36	AZA	0	NA	AZA	48	NA	NA	Va
	NMOSD (#48)	F/53	Pred, MMF	0	NA	Pred, AZA	24	NA	NA	Ia
	NMOSD (#53)	M/55	Pred, CyP; AZA	0.2	NA	Pred, AZA	61	NA	NA	Ia
	NMOSD (#58)	F/41	Pred, AZA	0.2	NA	AZA	90	NA	NA	I
	NMOSD (#61)	F/50	Pred, MMF	0.14	NA	MMF	84	NA	NA	Vb
SINGLE RTX DOSE	NMOSD (#5)	F/49	AZA; MMF	0.33	0	MMF	180	0 and 1	0 and 0.5	Ia
	NMOSD (#7)	F/35	Pred, AZA; MMF	0.11	0	Pred, MMF	117	1	7	1b
	NMOSD (#8)	F/36	Pred, AZA; MMF	0.9	0	Pred	96	1	5	III
	NMOSD (#9)	F/39	Pred, AZA	0.5	0	Pred, AZA	24	0 and 1	0 and 2	Ia
>1 RTX DOSE	NMOSD (#1)	F/48	Pred, MMF	1.02	0.09	RTX	59	14	4	Va
	NMOSD (#2)	F/34	Pred, AZA	1.73	0	RTX	21	10	2 and 11	Va
	NMOSD (#3)	M/46	Pred, AZA	0.89	0	RTX	41	12	13	Ia
	NMOSD (#4)	F/14	Pred, AZA, MMF	1.92	0	RTX	38	11	3 and 6	Va
	NMOSD (#6)	F/29	Pred, CyP, AZA, MTX	2.28	0.625	RTX	42	12	0.5	Ia
DISEASE CONTROLS	Migraine	M/29	None	NA	NA	None	44	NA	NA	Va
	Migraine	F/31	None	NA	NA	None	72	NA	NA	II
	Migraine	M/76	none	NA	NA	None	320	NA	NA	Ia
	NMDAR-Ab-E	F/31	Pred, AZA	NA	NA	Pred	58	NA	NA	Ib
	NMDAR-Ab-E	F/19	Pred	NA	NA	Pred	4	NA	NA	Va
	NMDAR-Ab-E	F/31	Pred	NA	NA	None	8	NA	NA	Ia
	NMDAR-Ab-E	F/29	Pred	NA	NA	None	27	NA	NA	Ia
	NMDAR-Ab-E	F/25	Pred	NA	NA	None	21	NA	NA	Va
	NMDAR-Ab-E	F/18	Pred	NA	NA	None	35	NA	NA	Ia
	NMDAR-Ab-E	F/21	AZA, MMF	NA	NA	Pred	222	NA	NA	Ib
	LGI1-Ab-E	M/55	None	NA	NA	None	48	NA	NA	Ia
	LGI1-Ab-E	F/71	none	NA	NA	None	8	NA	NA	Va
	GAD-Ab-E	F/66	None	NA	NA	None	12	NA	NA	Ib
	GlyR-Ab-E	F/56	Pred	NA	NA	Pred	36	NA	NA	Ib

Supplementary Table 2. Clinical characteristics of patients undergoing cervical lymph node fine needle aspirations (FNA). FNAs were performed in nine NMOSD patients treated with RTX, and five NMOSD patients naïve to RTX (overall, sampled at a median of 59 months into their disease; range 21-180). Also, FNAs were performed in 14 patients with other autoimmune and non-autoimmune neurological conditions. FNA was performed before and after RTX in two patients (NMOSD#5, NMOSD#9) and at two time-points after RTX in two other patients (NMOSD#2, NMOSD#4). From these four patients, FNA were from the same lymph node. Overall, cervical lymph node anatomical levels I, II, III and V were sampled. ARR = annualised relapse rate; AZA = azathioprine; CyP = cyclophosphamide; FNA = fine needle aspiration; GAD-Ab-E = glutamic acid decarboxylase antibody encephalitis; GlyR-Ab-E = glycine receptor antibody encephalitis; IT = immunotherapies; LGI1-Ab-E = leucine rich glioma inactivated 1 antibody encephalitis; MMF = mycophenolate mofetil; MTX = mitoxantrone; NMDAR-Ab-E = N-methyl-D-aspartate receptor antibody encephalitis; NMOSD = Neuromyelitis Optica spectrum disorders; Pred = prednisone; RTX = Rituximab.

			Heavy chain				Light chain			
Patient	Tissue	B cell subset	Isotype	V gene	J gene	Mutations	Type	V gene	J gene	Mutations
5	PBMC	Naïve	IgM	IGHV4-34*01	IGHJ5*02	0	Lambda	IGLV3-16*01	IGLJ1*01	0
58	PBMC	Naïve	IgM	N/A	N/A	N/A	Kappa	IGKV4-1*01	IGKJ4*01	1
58	PBMC	Naïve	IgM	N/A	N/A	N/A	Kappa	IGKV3-11*01	IGKJ4*01	0
58	PBMC	Naïve	IgM	N/A	N/A	N/A	Kappa	IGKV1-39*01	IGKJ3*01	0
53	PBMC	DN	IgM	IGHV3-30*18	IGHJ4*02	0	Kappa	IGKV3-15*01	IGKJ3*01	1
53	PBMC	IgD$^+$ mem	IgM	IGHV3-33*01	IGHJ4*02	5	Lambda	IGLV2-14*01	IGLJ1*01	3
53	dCLN	IgD$^+$ mem	IgM	N/A	N/A	N/A	Lambda	IGLV3-1*01	IGLJ2*01	11
5	dCLN	IgD$^-$ mem	IgG	IGHV4-34*01	IGHJ6*03	17	Lambda	IGLV3-19*01	IGLJ3*02	17
53	PBMC	IgD$^-$ mem	IgM	IGHV3-48*02	IGHJ2*01	14	Kappa	IGKV3-20*01	IGKJ2*01	2
58	PBMC	IgD$^-$ mem	IgM	IGHV3-30*18	IGHJ4*02	14	Kappa	IGKV2-28*01	IGKJ5*01	19
58	dCLN	IgD$^-$ mem	IgG	IGHV3-30*04	IGHJ6*02	10	Kappa	IGKV4-1*01	IGKJ3*01	10

Supplementary Table 3. Characteristics of AQP4-specific B cell receptor sequences retrieved from single B cell cultures. The table shows the patient number, tissue from which the B cell subset (naïve [CD27$^-$IgD$^+$]; double negative [DN; CD27$^-$IgD$^-$]; IgD$^+$ memory [mem; CD27$^+$IgD$^+$]; IgD$^-$ memory [CD27$^+$IgD$^-$]) was isolated and characteristics of both the heavy chains (isotype detected in culture, predicted V and J gene alleles and total number of variable region mutations; www.imgt.com) and light chains (lambda or kappa; predicted V and J gene alleles and total number of variable region mutations; www.imgt.com). From four B cells, heavy chains were not amplified (N/A). AQP4 = aquaporin-4; dCLN = deep cervical lymph node; DN = double negative; IGH = immunoglobulin heavy chain; IGL = immunoglobulin light chain; IgG = Immunoglobulin G; IgM = Immunoglobulin M; K = kappa; L = lambda; PBMC = peripheral blood mononuclear cells.
